# Hospital Expenditure.—The Commissariat

**Published:** 1895-11-30

**Authors:** 


### The Institutional Workshop.

### HOSPITAL EXPENDITURE.—THE COMMISSARIAT.

### VIII.—THE NURSES' TABLE—LONDON.

The standard of food for the nurses' table has risen in nearly all large institutions of late years until it may be said to represent in the best hospitals the ideal diet for women engaged in active work. The bill of fare quoted in the previous number reveals the enormous advance in such matters over the old style of provisioning. In the first place, the nitrogenous element is conspicuous. Meat, or a substitute for meat, fish, eggs, &c., is provided three times daily, besides cheese. Secondly, variety is aimed at; the dishes admit of modification and seasonal changes, and the old rigid time-table system of beef, mutton, and gammon hock is coming to be entirely discredited. Thirdly, some allowance is made for differences of taste and digestion, and it is no longer a crime to refuse fat bacon or prefer cocoa to tea. These changes have not been carried out without some additional cost, but among all the items of increased expenditure which come under the notice of committees and subscribers we think none can be regarded with feelings of more unmixed satisfaction. The old plan vividly present to the memory of many, under which a good proportion of the nurse's scanty wages went in supplementing the coarse diet provided, was a discredit less to individual institutions than to the entire hospital system. It would be sanguine to conclude that the improvement has already become universal, for, as an experienced matron declares, "there will always be an average of stale eggs and of monotony in the way of roast and boil and milk puddings," but public opinion has now set in the right direction, and must in the end succeed in drawing all the laggards into line.

An examination of the cost of the nurses' board in some of the leading London hospitals reveals a curious variety of system and expenditure co-existing with a high standard of general quality.

University College Hospital stands in the unique position of boarding none of the nurses. They belong exclusively to the Anglican Sisterhood of All Saints, Margaret Street, and are boarded entirely under the direction of the Superior. The sum paid to the sisterhood for the nursing of the hospital is inclusive of an allowance of 10s. a head for the board of the 87 nurses. This sum, amounting to £2,262, would, it may be observed, be more properly entered in the accounts according to the uniform system under the head of Provisions—Board of Nurses. It amounts to as much as an additional £12 for provisions on each occupied bed, and raises the total cost of provisions per occupied bed for the year from £18 10s. to nearly £31. The nurses' share of the total cost of provisions amounts to 39 per cent. It must be remembered that, though this seems a large amount for the hospital to pay out for this purpose, it is by no means an excessive sum for the sisterhood to receive. Out of this 10s. a week has to be paid not only fuel, lighting, kitchen, wear and tear and rent of premises, but the board and wages of a certain number of servants employed in the preparation and serving of the meals. None of these items are charged in the cost of provisions for the nurses in other hospitals where the victualling is done on the premises, or in nursing homes attached to the hospital. The first-named items are not appreciably added to in a large hospital by the additional food prepared for the nurses; the sisterhood expends more on fuel, &c., by boarding 87 extra persons, but the hospital probably saves little. A definite deduction should, however, be made for the board and wages of extra servants who would certainly be required if the nurses boarded in the hospital. Six maids at £30 a year inclusive, or a total of £180, would be a fair allowance; and this reduces the cost of the actual nurses' board to £2,082, or £11 on each occupied bed.

An almost similar reckoning of the cost of the nurses' board is made by Westminster Hospital, with this conspicuous difference, that the hospital instead of disbursing that amount for boarding the nurses off the premises, receives it for the cost of their food, or rather deducts it from the lump sum paid for the nursing of the hospital. The cost of the nurses' food, minus all the outside expenses which we have pointed out, is reckoned £25 a year, or 9s. 7d. a week, a sum relatively far higher, when these items are considered, than that paid at University. Owing to the small number of nurses boarded (36 out of a total staff of 55), the cost of nurses' provisions is but £900, or £5 per occupied bed, and the proportion of nurses' board to that of the whole establishment is but 21 per cent. Nothing could illustrate more forcibly the intricacies of hospital finance than this result, whereby the highest average for the board of the individual nurse works out by far the lowest percentage of total cost. In other words, Westminster, paying half as much again as some other hospitals for the board of each nurse, expends half as little in proportion in the board of them all.

The cost of boarding the 275 nurses in the nurses' home attached to the London Hospital is stated in the annual report to be £6,205, this sum representing merely the cost of provisions. The board of 14 servants is, however, included in this amount, and deducting for each of these a sum of 6s. a week, the cost of boarding the nurses works out at about 8s. 4d. a head per week. This amounts to £9 a year per occupied bed, or about 33 per cent. of the total cost of provisions.

At the Royal Free Hospital the average weekly cost of nurses' board is from 7s. 7d. to 8s. a head. The cost of boarding the 40 nurses amounts to £7 a year to each occupied bed, and the percentage is 26 of the total cost of provisions.

The Nightingale Home, where 45 inmates are boarded at a weekly cost of 8s. 6d. a head, is slightly above this average in numbers and cost.

At St. Thomas's nursing home the total amount assigned to nurses' provisions in the annual report is £2,778. Again, however, allowance must be made for the boards of 35 wardmaids and servants, besides partial board to the scrubbers. Deducting for the servants at the lower rate of 6s. a week per head, the cost of nurses' board works out at slightly under 8s. 6d. a head, or about the same as at the Nightingale Home. The yearly cost of nurses' provisions (exclusive of servants) at St. Thomas's is rather under £6 on each occupied bed, and the percentage of total cost of provisions for the hospital is 21. This low percentage is due to the fact that the probationers are all boarded under a separate account not chargeable to the hospital in the Nightingale Home. If the whole cost of boarding both nurses and probationers were reckoned the yearly charge on each bed for nurses' provisions would amount to £10, and the cost of their board would rise to 33 per cent. of the total cost of provisioning the hospital.

At St. Bartholomew's 240 nurses and 71 servants are boarded at a total cost of £5,460. Deducting at the rate of 6s. a week each for the servants, the cost of the nurses works out at something under 7s. a week. The yearly cost of the nurses' board is £8 per occupied bed, and the percentage of the total cost of provisions is 30 per cent.

At Guy's, the board of 172 nurses and 35 servants reaches the total of £3,239. The cost per head is about 6s. 4d. a week. The yearly cost of the nurses' board amounts to £6 15s. on each occupied bed, and the nurses' share of the total cost of provisions is 28 per cent.

It may be well to mention with all these calculations that the figures in the published reports do not always give the exact cost only of all provisions consumed during the year. Small as the storage accommodation is in most hospitals, few of them live entirely from hand to mouth; and a large consignment of dry goods at the end of a financial year may add something to that particular average and ease the following year's statement in a corresponding fashion.

The deduction of 6s. a head for each servant, though not strictly accurate in each case, is, it is believed, sufficiently near the mark to give a fair working average.

